# From blue November to broader diagnosis: The Youden index to evaluate the performance of any diagnostic tests

**DOI:** 10.1016/j.clinsp.2025.100804

**Published:** 2025-10-08

**Authors:** Paulo Sergio Panse Silveira, Flavio Trigo Rocha, Joaquim Edson Vieira, Jose Oliveira Siqueira

**Affiliations:** a Departamento de Patologia da Faculdade de Medicina da Universidade de São Paulo, SP, Brasil; b Disciplina de Urologia, Departamento de Cirurgia, Faculdade de Medicina da Universidade de São Paulo, SP, Brasil; c Seção de Disfunção Miccional em Urologia, Hospital Sírio-Libanês, São Paulo, SP, Brasil; d Disciplina de Anestesiologia, Departamento de Cirurgia, Faculdade de Medicina da Universidade de São Paulo, SP, Brasil; e Faculdade Israelita de Ciencias da Saúde Albert Einstein, São Paulo, SP, Brasil

**Keywords:** Prostate-Specific Antigen; Predictive value of tests; Sensitivity and specificity; Diagnostic techniques and procedures; Statistics as topic

## Abstract

**Objective:**

This is a methodological study. The goal is to describe and implement statistical tests based on the Youden index to evaluate the performance of diagnostic tests, using Prostate-Specific Antigen (PSA) as the primary example and including additional diagnoses to illustrate how these evaluations can be generalized.

**Methods:**

Quantitative analysis using the Youden index was applied to assess diagnostic test performance across three different experimental designs: a single condition, two independent conditions (between-groups), and two dependent conditions (within-group), revisiting 2 × 2 tables from previous studies.

**Results:**

The Youden method combines sensitivity and specificity into a single index and requires only a 2 × 2 contingency table summary, incorporating both point estimates and confidence intervals. This allows for the evaluation of many studies where raw data are unavailable.

**Conclusion:**

PSA seems insufficient for effective prostate cancer screening, despite numerous efforts over decades claiming improvements in sensitivity, specificity, or diagnostic capability. However, the statistical method presented here can be applied to any symptom, sign, or laboratory test, current or future. By providing open-source code, the authors aim to bridge the gap between statistical methods and their practical application, improving diagnostic processes. The R package and other supplemental materials to replicate this study are available on Harvard Dataverse at https://doi.org/10.7910/DVN/5QTMBW.

## Highlights

• Many efforts to improve PSA tests have led to minimal or no significant progress.
• Youden index is a statistical method for evaluating diagnostic tests.
• It also provides a rule of thumb: sensitivity + specificity >1 is required for a diagnostic test.
• Prevalence and individual probability of disease are interchangeable parameters for clinical practice.
• This statistical method can be applied to any symptom, sign, or laboratory test, current or future.

## Introduction

This study is primarily methodological, centered on the Prostate-Specific Antigen (PSA) as a key case study. The authors emphasize the practical implementation of these statistical methods, reifying their application in clinical decision-making. The approach that the authors present is adaptable for evaluating any symptom, sign, or laboratory test, whether already in use or to be developed in the future.

In particular, the authors focus on presenting the Youden index as a concrete tool for clinical decision-making. Although the index was proposed in 1950^1^ and further refined in 2015,^2^ its practical use remains limited. In most cases, its application is restricted to identifying the optimal cut-off point in ROC curve analyses. However, the Youden index can also be used to assess whether a diagnostic exam meets the minimum performance criteria to be considered useful, and to statistically compare the performance of different tests when evaluating potential diagnostic improvements. In this study, the authors operationalize these applications through detailed examples to facilitate their generalization for use in both clinical and research contexts.

Prostate cancer is the most frequently diagnosed malignancy in men, accounting for 26 % of new cancer cases and being the second leading cause of cancer-related deaths, responsible for 11 % of mortality, following lung cancer. The lifetime risk of developing microscopic prostate cancer is about 30 %, with a clinical disease probability of 10 % to 11 %, and the risk of dying from it ranges from 2.5 % to 3 %.^3^^,^^4^

The PSA case is particularly interesting because population screening through digital rectal exams and PSA blood tests is promoted by the Blue November campaign. Initiated in Australia in the 1980s, the Blue November campaign aims to raise awareness about prostate cancer and encourage early detection. Over time, it has evolved and been adapted across various countries, incorporating different strategies.^5^^,^^6^

Routine screening for Prostate-Specific Antigen (PSA) levels is a controversial issue. In the UK, it is claimed that while screening may reduce prostate cancer mortality, it can also lead to unnecessary treatments.^7^ The American Cancer Society (ACS) advocates for informed decision-making between men and their doctors regarding screening, emphasizing the need to consider uncertainties and risks.^8^ Since 2023, Brazil’s Ministry of Health, following WHO guidelines, has advised against screening asymptomatic men, which contrasts with other recommendations on the same website.^9^, ^10^, ^11^, ^12^, ^13^ PSA alone may not be sufficient for prostate cancer detection.^14^ Recent guidelines suggest a risk-adapted approach for men over 50 at increased risk, promoting magnetic resonance imaging to avoid unnecessary biopsies^15^; however, it is unfeasible for population-wide screening. Major urological societies recommend screening only for men with low comorbidities and a reasonable life expectancy.^16^ The American Urological Association and the Brazilian Society of Urology do not recommend screening for men under 40 or over 70 (or with less than 10-years of life expectancy), suggest biennial screening for men aged 55‒69 based on shared decision-making, and do not actively discourage screening for high-risk men aged 40‒54.^17^ Prostate cancer management, including screening, should focus on reducing mortality and preserving quality of life by minimizing overdetection from the PSA test. While individualized screening based on baseline PSA levels is valid,^18^ widespread screening of asymptomatic individuals can lead to overdiagnosis and harm. Understanding diagnostic reasoning and its biases is essential to promoting evidence-based changes in medical practices.

Instead of focusing on the controversy surrounding population screening, which has been extensively covered in the literature, the validity and quality of the available diagnostic instruments is what truly must be addressed. We apply this reasoning to highlight the limitations of the PSA test using the Youden index. The improvements in sensitivity and specificity observed in many studies are merely point estimates, necessitating statistical tests to determine whether these improvements are significant.

Although Youden’s index (*J*) is well established in the literature,^1^ it remains underutilized by physicians in daily practice. The index states that the sum of sensitivity (*se*) and specificity (*sp*) minus 1 must be greater than 0, summarized as J=se+sp−1, with a maximum value of 1. If the sum is below 0, the diagnostic test is considered useless; if it exceeds 0 but not significantly, uncertainty remains. The closer the value is to 1, the better the test.

The implementation described here provides the statistical test to verify whether an exam has a Youden index significantly greater than zero. When comparing multiple tests (e.g., the performance of total PSA and free-to-total PSA alternatives), it is essential not only to verify the validity of each individual test but also to perform comparisons using both between-groups (independent groups) and within-group (same individuals measured by two exams) designs.

By making these procedures available, we aim to assist healthcare professionals in assessing the diagnostic quality of exams used in their clinical practice.

## Method

### Background

This text explains the diagnostic value of a test through the Youden index, using the notation in Table 1. The table relates interactions between test (*T*, which is an observable symptom, a signal detected by physical examination, a laboratory result, or an imaging diagnosis), and disease (*D*, which is any patient status, such as the presence of a disease, the occurrence of death, or the existence of a morbid condition). This relation has concordant results (counts in cells *a* or *d*) and disagreements (counts in cells *b* or *c*).

**Table 1.** *D_+_* and *D_-_* represent the presence or absence of a disease or patient condition, respectively. *T_+_* and *T_-_* denote the occurrence of a positive or negative result from any symptom, signal, or test. Along the main diagonal, *a* and *d* indicate the counts or proportions of agreement between *D* and *T*, while along the secondary diagonal, *b* and *c* indicate the counts or proportions of disagreement between *D* and *T*.

	***D_+_***	***D_-_***	**Total**
***T_+_***	*a*	*b*	*a+b*
	True positive	False positive	
***T_-_***	*c*	*d*	
	False negative	True negative	*c+d*
**Total**	*a+c*	*b+d*	*a+b+c+d*

The key concepts are:

• Prevalence (*p*) is the probability or proportion of diseased patients.
• Sensitivity (*se*) is the “the probability of a positive test provided that the patient is diseased”, *a*/(*a* + *c*).
• Specificity (*sp*), similarly, corresponds to “the probability of a negative test provided that the patient is not diseased”, *d*/(*b* + *d*).
• Positive (*PPV*) and negative (*NPV*) predictive values, which are the primary interest for physicians facing a patient whose status (diseased or not diseased) is unknown.

The distinction between sensitivity or specificity (which reflect the laboratory quality of a test) and positive or negative predictive values (which relate to the diagnostic quality for a patient) must not be confused. As shown below, even a highly sensitive and specific test may not yield a high probability of diagnosing or excluding a disease based on the result. Since physicians typically prefer to think in terms of the presence of disease, the authors applied the complement of *NPV* (1−NPV) throughout the results, which represents the remaining probability of disease when the test result is negative.

Since sensitivity and specificity are unaffected by prevalence, any number of healthy and diseased subjects (gold standard) can be recruited to compose the contingency table. However, determining *PPV* or *NPV* is valid only for a contingency table that reflects the prevalence of the population from which the patients are drawn. In many publications aiming to improve a laboratory test report “improvements” in *PPV* and *NPV* as if they were indicators of test performance, which is biased by patients predisposed to the disease under study, leading to confusion between the test’s laboratory performance and its utility in clinical diagnosis.

### Simulation

A simulation illustrates how the concepts discussed in the previous section (R script available in the supplemental material) ‒ *se, sp, PPV*, and *NPV* ‒ vary across different prevalence levels. Scenarios with 500,000 hypothetical 2 × 2 tables demonstrate that prevalence does not influence sensitivity or specificity, but does affect predictive values, which are crucial for patient diagnosis in clinical practice.

### Youden’s index

This study demonstrates how to replicate and implement the Youden index and related statistical tests (*J*).^1^ The index measures test performance by comparing true (positive or negative) results with false (positive or negative) results, with the goal of having more correct results than incorrect ones (Table 1).

The original author of this index also highlighted that false negatives are especially problematic when delayed treatment can affect the course of a disease, while false positives can lead to the misuse of resources meant for genuinely diseased patients. Determining which type of diagnostic error is more important is a clinical decision, not a statistical one.

### One-sample test of the Youden index

In a single-condition design, it is essential to determine how well a test performs, regardless of the disease’s prevalence. The Youden index serves as a summary measure of the test’s overall diagnostic capability. It statistically determines whether an exam can be considered a valid diagnostic test when the confidence interval of the Youden index is greater than zero. This involves a one-sided statistical analysis with the following hypothesis:

(1) $$\begin{array}{c} H_{0}:J\leq 0\\ H_{1}:J>0 \end{array}$$

The statistical test was implemented as an R function (eiras2x2::onesample.Youden).

The core of its implementation lies in the computation of the standard error to derive the confidence interval, a method originally developed by Youden^1^ and later refined by Chen et al. in 2015.^2^

### Independent samples test of Youden indices (between groups)

When a new test is developed as a proposed improvement over a reference test, and both tests are applied to two groups of patients, this statistical comparison determines whether the new test performs significantly better than the existing one.

Inferential statistics tests the null hypothesis:

(2) $$\begin{array}{c} H_{0}:J_{1}=J_{2}\\ H_{1}:J_{1}\neq J_{2} \end{array}$$

The experiment can be conducted with two independent groups of subjects. It was implemented using both the original approach^1^ and the modified approach described above.^2^

### Dependent samples test of Youden indices (within-group)

The within-group design accounts for the agreement between the two tests applied to the same subjects to compute the standard error, aiming to achieve greater statistical power. Chen et al.^2^ used Cohen’s kappa as a measure of agreement, but our implementation opts for Gwet’s AC1 or Holley and Guilford’s G, which are considered more appropriate estimators of agreement.^19^

### Strategy of analysis

In a hierarchical approach, each test is first evaluated using the single-condition design to determine if it meets diagnostic criteria. Once confirmed, comparisons between the two tests are made, applying independent or dependent designs as appropriate.

### Supplementary materials and data availability

Supplementary material is available at the Harvard Dataverse at https://doi.org/10.7910/DVN/5QTMBW, including the R package eiras2x2 that contains the functions used in this study, the equations corresponding to the methods described here, and scripts that demonstrate how to use the package to replicate all the figures and tables presented.

The “Youden Index Calculator”, a small web-based tool, is also provided at http://dataverse.harvard.edu/api/v1/access/datafile/11720167 for direct access. It works on both computers and mobile devices, and requires downloading the youden.html file to be opened locally in a web browser. It allows users to evaluate diagnostic performance and compute post-test probabilities based on prevalence or the clinician’s prior estimate.

## Results

### Simulation

It is asserted that sensitivity (*se*) and specificity (*sp*) are not influenced by disease prevalence, but positive and negative predictive values are.^20^ We simulated 500,000 2 × 2 tables with 500 observations each. Prevalence values between 1 % and 99 % were randomly assigned, and sensitivity and specificity values were generated based on predefined Youden index (*J*) values in 10 % intervals. For example, for *J* = 0*.*4, valid pairs included (se=0.70,sp=0.70),(se=0.57,sp=0.83), and others. From these, *PPV* and the complement of *NPV* were calculated.

**Fig. 1:**
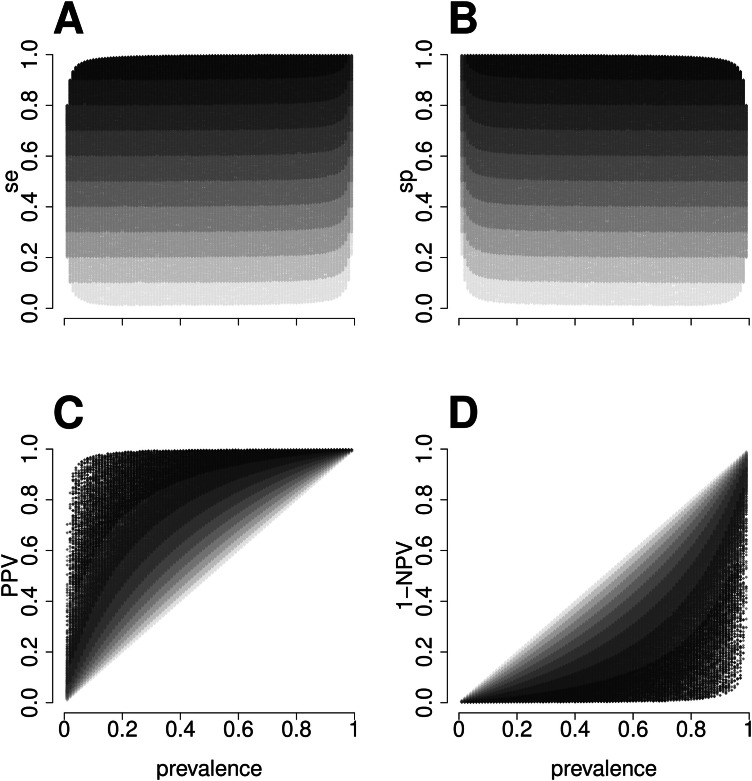
Simulation of 500,000 2 × 2 tables (*n* = 500) with the Youden index ranging from 0 *<J<* 1 in intervals of 10 % (light to dark gray). The figure shows that sensitivity (*se*) and specificity (*sp*) do not depend on prevalence. However, the probability of disease is affected by prevalence, increasing more with positive test results (Positive Predictive Values, *PPV*) and decreasing more with negative test results (complement of the Negative Predictive Values, 1*−NPV*) as the value of *J* increases.

In Fig. 1, higher *J* values correspond to higher *se* and *sp*, forming horizontal bands (Fig. 1A and B). *PPV* and complement of *NPV*, influenced by prevalence, create crescent-shaped bands moving away from the bisector as *J* increases (Fig. 1C and D).

### Youden’s index

#### Prevalence and pre-test probability: Interchangeable concepts

Although prevalence is an epidemiological concept, it also applies to individual patients as pre-test probability. The physician’s intuition for an individual patient is equivalent to population prevalence in estimating disease probability, placing the patient in a subpopulation with specific symptoms and signs where the diagnosis prevalence is higher. For example, a patient with headaches and a family history of hypertension belongs to a subpopulation with a higher prevalence of hypertension. From that, the test designed for the population also applies to individuals, and they must adjust the physician’s belief in a diagnosis (pre-test) when the result is positive or negative (post-test probabilities, or the updated belief).

#### Sensitivity and specificity: The key to exclusion and confirmation in diagnostic testing

**Fig. 2:**
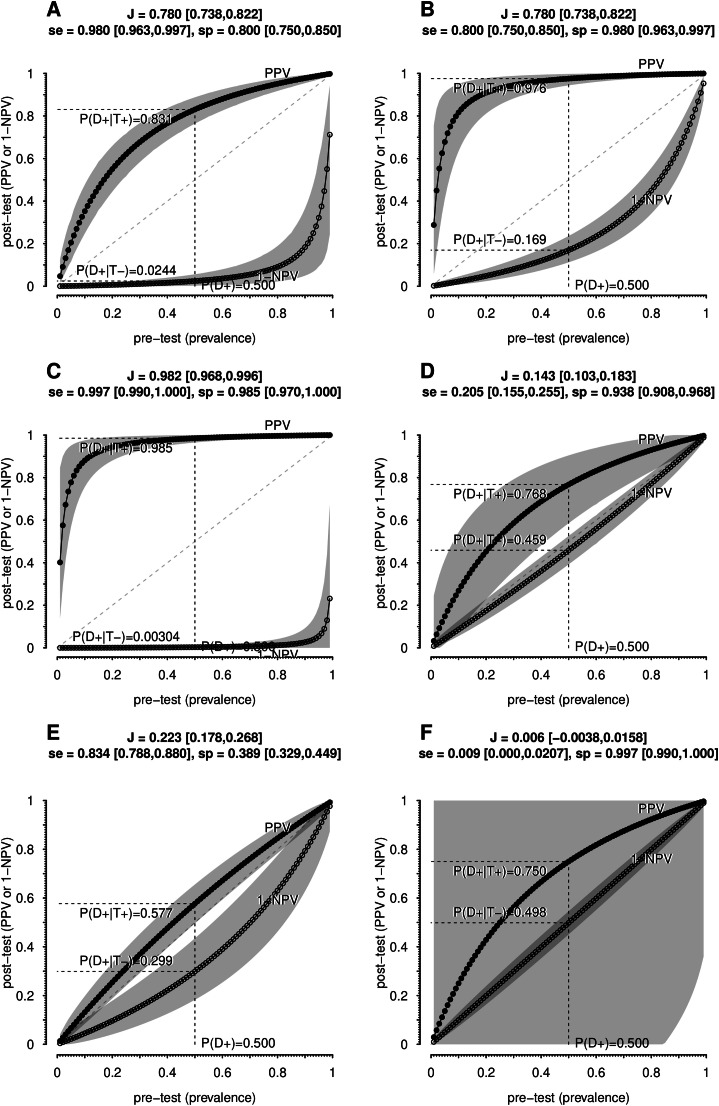
Examples of diagnostic exams with varying sensitivity and specificity values. It is illustrated with updated probabilities of disease (pre-test) with an initial suspicion of 50.0 % after a positive test result (*PPV*) or negative test result (1 *− NPV*). Shadows are 95 % confidence bands (*n* = 500). (A and B) Hypothetical exams with equal values of Youden index (*J*) switching sensitivity (*se*) and specificity(*sp*); (C) Estimated values for ELISA for HIV detection (França et al., 2018); (D, E and F) Prostate detection assessed by seric PSA varying the cutoff points, respectively 4.1, 1.1, and 10.1 ng/mL (Thompson & Ankerst, 2007).

Sensitivity and specificity bring distinct attributes in clinical situations. For instance, two tests with moderately high *J* updates diagnoses differently depending on sensitivity and specificity. Fig. 2A and B illustrate a pre-test estimate of 50.0 %. For a test with se=0.98,sp=0.80, a positive result raises the probability to 83.1 %, a gain but not a strong confirmation, while a negative result lowers it to just 2.4 %. Conversely, for se=0.80,sp=0.98, the same pre-test probability is updated to 97.6 % with a positive result, but only to 16.9 % with a negative result, which may not be sufficient to rule out the diagnosis.

Ultimately, a highly sensitive test is most useful for ruling out a diagnosis when negative, while a highly specific test is best for confirming a diagnosis when positive.

#### The fallacy of almost perfect tests: The illusion behind high sensitivity and specificity

The trap for the physician occurs when there is no diagnostic suspicion, and a test is performed just to rule out a disease. For example, the sensitivity of the Enzyme-Linked Immunosorbent Assay (ELISA) for HIV detection was estimated at 99.7 % and specificity at 98.5 %,^21^ as depicted in Fig. 2C. From an initial pre-test probability of 50 %, a positive result updates the probability of disease to 95.5 %, and a negative result updates it to 0.3 %. It seems like an excellent test.

However, sensitivity and specificity close to 100 % can be misleading. Without the patient being from a risk group, the doctor might adopt the general population estimate as the probability that the patient is HIV positive. The prevalence of HIV in the general population is 0.24 %. Under this assumption, a negative test practically rules out the infection (1−NPV=0.000732%), but a positive test provides only a small probability of HIV infection (PPV=13.7%)! Thus, it is necessary to exercise caution before confirming the diagnosis, and more tests, especially specific ones, may need to be requested, such as Western Blot or Nucleic Acid Tests.

On the other hand, if the patient belongs to a subpopulation with higher HIV prevalence, such as intravenous drug users in Brazil (23.1 %)^22^ or female sex workers in Cambodia,^23^ the interpretation of the test dramatically changes. The same test now gives PPV=95.2%and1−NPV=0.0914%.

These results may seem counterintuitive to those unfamiliar with this type of evaluation. Many assume that a highly sensitive and specific test guarantees diagnostic accuracy. However, this overlooks the importance of patient context, clinical history, and, critically, the prevalence of the population from which the patient comes.

#### The stagnation of PSA testing: Decades of adjustments without substantial diagnostic improvement

The Prostate-Specific Antigen (PSA) test, commonly used for prostate cancer screening, has varying sensitivity and specificity depending on the PSA cut-off used. The traditional 4.0 ng/mL cut-off has been debated for producing false positives, leading to unnecessary biopsies and anxiety. Some guidelines suggest alternative cut-offs to improve accuracy.

For instance, Thompson & Ankerst^24^ report *se* = 20*.*5 % and *sp* = 93*.*8 % for a 4.1 ng/mL PSA cut-off. With a pre-test probability of 50 %, the present results show a PPV of 76.8 %, which may not justify a biopsy, and a negative result reduces the probability to 1 − *NPV* = 45*.*9 %, offering little reassurance (Fig. 2D). These authors also proposed alternative cut-off points of 1.1 ng/mL (*se* = 83*.*4 %*, sp* = 38*.*9 %) or 10.1 ng/mL (*se* = 0*.*9 %*, sp* = 99*.*7 %). Lowering the cut-off point is an attempt to increase sensitivity, which updates the initial 50.0 % to *PPV* = 57*.*7 % and 1 − *NPV* = 29*.*9 % (Fig. 2E); raising it to 10.1 ng/ml improves PPV (75.0 %) but sacrifices sensitivity and does little to rule out cancer (1 − *NPV* = 49*.*8 % Fig. 2F). There is an additional problem: the gray shadows representing 95 % confidence bands computed for a hypothetical study with 500 patients spans the entire area of the graph, making the estimate of 75.0 % meaningless in non- informative interval.

Each adjustment in sensitivity and specificity seems to solve one problem while introducing another, highlighting the need for statistical testing to assess the effectiveness of these changes in PSA testing. The results below demonstrate that many efforts to improve PSA tests and their variants have led to minimal or no significant progress.

### Statistical tests

We operationalize the statistical tests through examples, including the evaluation of a single condition (to verify whether a test qualifies as diagnostic) and comparisons between two conditions (to compare the performance of different tests). The examples are based on data from Chen et al.^2^ on bladder carcinoma and from Erdogan et al.^25^ and Recker et al.^26^ on PSA.

Our contributions include (1) Rewriting the equations for clarity, (2) Stating the null hypotheses (expressions 1 and 2), and (3) Implementing all procedures in R functions (see Supplemental Material for details). The main functions are *onesample.Youden* to test the null hypothesis in expression 1, and *twosample.Youden* to compare the relative performance of two diagnostic tests, considering expression 2, for both between-groups and within-group comparisons.

### One-sample test of the Youden index

Before comparing diagnostic exams, each candidate must first be tested individually. This one-sample test of the Youden index verifies whether an exam qualifies as a diagnostic test and is included as a preliminary analysis in the following sections.

Chen et al.^2^ proposed improvements to Youden’s test.^1^ Their example compares immunoCyt™ and cytology performed by a pathologist for diagnosing bladder carcinoma. Both exams are diagnostic tests, showing that *J >* 0 according to the original Youden statistic as well as the modification by Chen et al. (Table 2, upper panel).

**Table 2.** Reproduction of Chen et al. (2015) data for the estimation of exams for bladder carcinoma: immunoCyt™ and Cytology. For each test, sensitivity (*se*), specificity (*sp*), Youden index (*J*), and *p* value were computed using the R function *onesample.Youden* testing $H_{0}:J\;\leq \;0$. For the difference between tests, 95 % confidence interval was computed by the R function *twosample.Youden* testing *H*_0_: $J_{2}-J_{1}=0$.

	immunoCyt™		Cytology	
	*D_+_*	*D_-_*	*D_+_*	*D_-_*
*T*_+_	85	51	41	12
*T*_-_	16	182	13	24
*se*	84.16 [77.04, 91.28]		75.93 [64.52, 87.33]	
*sp*	78.11 [72.80, 83.42]		66.67 [51.27, 82.07]	
*J* (Youden 1950)	0.6227 [0.5482, 0.6972]		0.4259 [0.2651, 0.5867]	
	***p*****=2.91**^**.**^**10**^**–43**^		***p*****=6.61**^**.**^**10**^**–6**^	
*J* (Chen 2015)	0.6227 [0.5628, 0.6826]		0.4259 [0.3089, 0.5430]	
	***p*****=6.67**^**.**^**10**^**–66**^		***p*****=1.08**^**.**^**10**^**–9**^	
*J_2_ - J_1_* (Youden 1950)	−0.1968 [−0.4080, 0.0144]		***p*****=0.06784**	
*J_2_ - J_1_* (Chen 2015)	−0.1968 [−0.3534, −0.0401]		***p*****=0.01382**	

Verifying the quality of each test is necessary, regardless of whether the study design is between-subjects or within-subjects. Therefore, in all the tables that follow, the diagnostic tests are routinely evaluated.

The following have sufficient performance to be considered diagnostic tests:

• ELISA and ELISPOT methods for tuberculosis detection^2^ Table 3, upper panel.

**Table 3.** Reproduction of Chen et al. (2015) data for the estimation of exams for tuberculosis detection in a within-group design: all patients were tested with ELISA and ELISPOT methods. For each test, sensitivity (*se*), specificity (*sp*), Youden index (*J*), and p value were computed using the R function *onesample.Youden* testing $H_{0}:\;J\;\leq \;0$. For the difference between tests, confidence interval was computed by the R function *twosample.Youden* testing $H_{0}:\;J_{2}-J_{1}\;=\;0$.^a^.

	**ELISA**				**ELISPOT**		
	*D_1+_*		*D_1-_*		*D_2+_*		*D_2-_*
*T_1+_*	182		47	*T_2+_*	192		45
*T*_*1-*_	45		211	*T*_*2-*_	35		213
*se*	80.18 [74.99, 85.36]			84.58 [79.88, 89.28]			
*sp*	81.78 [77.07, 86.49]			82.56 [77.93, 87.19]			
*J*	0.6196 [0.5719, 0.6673]			0.6714 [0.6251, 0.7177]			
	***p*****=9.38**^**.**^**10**^**–102**^			***p*****=2.84**^**.**^**10**^**–126**^			
**Diseased group:**				**Control group:**			
	**ELISA**				**ELISA**		
**ELISPOT**	*T*_*1+*_	*T*_*1-*_	Total	**ELISPOT**	*T*_*1-*_	T_1+_	Total
*T*_*2+*_	181	11	192	*T*_*2-*_	207	6	213
*T*_*2-*_	1	34	35	T_2+_	4	41	45
Total	182	45	227	Total	211	47	258
Agreement (*AC_1_*)	0.926			0.945			
	Diff. *J*			0.05180 [−0.02293, 0.12654]			
	***p*****=0.1743**						

a Boxes are one option that can provide enough information to recover the partition between diseased and control groups: marginals are transported from the original tables. The number of patients with positive or negative results in both methods allows the reconstruction of 2 × 2 tables.
• PSA with different cutoffs for prostate cancer detection in two groups according to prostate volume^25^ shows that PV qualifies as a diagnostic test (with smaller volumes suggesting cancer), but it has low to moderate sensitivity and specificity (Table 4, upper panel).

**Table 4.** Analysis of Erdogan et al. (2020) data for the estimation of Prostate Volume (PV, mL) as a predictor of Prostate Cancer (PCa). For each test, sensitivity (*se*), specificity (*sp*), Youden index (*J*), and *p* value were computed using the R function *onesample.Youden* testing $H_{0}:\;J\;\leq \;0$. For the difference between tests, confidence interval was computed by the R function *twosample.Youden* testing $H_{0}:\;J_{2}-J_{1}\;=\;0$.

	PSA 2.5‒10.0 ng/mL			PSA 10.1‒30.0 ng/mL	
	PCa*_+_*	PCa*_-_*		PCa*_+_*	PCa*_-_*
PV < 43.5	29	13	PV < 61.5	21	10
PV > 43.5	19	83	PV > 61.5	5	31
*se*	60.42 [46.58, 74.25]			80.77 [65.62, 95.92]	
*sp*	86.46 [79.61, 93.30]			75.61 [62.46, 88.75]	
*J*	0.4688 [0.3625, 0.5750]			0.5638 [0.4327, 0.6949]	
	***p*****=1.94 ^.^ 10**^**–13**^			***p*****=7.46 ^.^ 10**^**–13**^	
*J_2_ - J_1_* = 0.0950 [−0.1060, 0.2961], ***p*****=0.3541**
• Total PSA (tPSA) and its variants aiming to improve prostate cancer diagnosis using free-to-total PSA ratios with cut-off points at 0.20 (f/tPSA020) and 0.15 (f/tPSA015).^26^ All exams under evaluation qualify as a diagnostic test (Table 5).

**Table 5.** Reproduction of Recker et al. (1998) data for the estimation of Prostate-Specific Antigen (PSA) tests: Total PSA (tPSA), free to total PSA (f/tPSA020) with a cut-off point at 0.20, and free to total PSA with a cut-off point at 0.15 (f/tPSA015). Patients were diagnosed with prostate cancer (*D*_+_) or benign prostatic hyperplasia (*D*_-_), and the PSA tests could result in positive (*T*_+_) or negative (*T*_-_) outcomes. Sensitivity (*se*), specificity (*sp*), Youden index (*J*), and *p* values were computed using the R function *onesample.Youden* testing $H_{0}:\;J\;\leq \;0$.

	**tPSA**		**f/tPSA020**		**f/tPSA015**	
	*D_+_*	*D_-_*	*D_+_*	*D_-_*	*D_+_*	*D_-_*
*T*_+_	61	93	61	74	48	39
*T*_-_	8	126	8	145	21	180
*se*	88.41		88.41		69.57	
	[80.85, 95.96]		[80.85, 95.96]		[58.71, 80.42]	
*sp*	57.53		66.21		82.19	
	[50.99, 64.08]		[59.95, 72.47]		[77.12, 87.26]	
*J*	0.4594		0.5462		0.5176	
	[0.3987, 0.5201]		[0.4835, 0.6088]		[0.4371, 0.5981]	
	***p*****=7.87**^**.**^**10**^**–36**^		***p*****=5.75**^**.**^**10**^**–47**^		***p*****=1.99**^**.**^**10**^**–26**^

A detailed description of these studies and the comparison between methods is provided below.

### Independent samples test of Youden indices (between groups)

The null hypothesis of equality between immunoCyt™ and cytology performed by a pathologist for diagnosing bladder carcinoma is not rejected using Youden’s original method, as the confidence interval includes zero ; however, it is rejected with Chen’s correction^2^ (Table 2, lower panel), since the difference *J*_2_ − *J*_1_ lies to the left of zero (i.e., *J*_1_ *> J*_2_, indicating that immunoCyt™ performs better than cytology). Therefore, Chen’s method is used throughout the remainder of the text.

A second example is the study by Erdogan et al.^25^ selected because it provides sufficient data to reconstruct the 2 × 2 tables of interest. As is often the case in similar studies, this information is presented in a convoluted and wordy manner. The central question in this example is whether prostate volume is a better predictor of prostate cancer than PSA, using biopsy diagnosis as the gold standard and dividing patients into two groups based on PSA concentration.

Patients were divided into two groups based on PSA levels (2.5–10.0 ng/mL and 10.1–30.0 ng/mL), with each group having a different prostate volume cutoff determined by ROC curves: 43.5 mL and 61.5 mL, respectively. In an attempt to improve specificity, the authors applied PSA density (PSAD), defined as the PSA/PV ratio, and the free-to-total PSA ratio (f/tPSA).

Since PSA, PSAD, and f/tPSA data are unavailable, the only possible analysis here is to compare patients, separated by PSA concentration, to assess if there is a difference in PV performance between the two groups.

The conclusion is that although PV qualifies as a diagnostic test (with smaller volumes suggesting cancer), it has low to moderate sensitivity and specificity (Table 4, upper panel). The test shows similar performance regardless of the PSA level used to divide the patients into groups (Table 4, lower panel). Since PV was assessed using ROC curves in the original article, but the raw data are unavailable, the authors’ claim that “PV was a significantly better indicator of PCa than PSAD and f/t PSA ratio in both groups” cannot be verified here.

Note, however, that the authors did not evaluate the values of PPV, as mentioned by the authors (nor NPV, which wasn’t mentioned but could be similarly calculated). These values should not be considered for samples that do not reflect the population’s prevalence. Instead, it is more informative to observe the range of values in the PPV and complement of NPV curves.

### Dependent samples test of Youden indices (within-group)

For the paired test, the example from Chen et al.^2^ is presented in two tables that are somewhat challenging to interpret. The key step is partitioning the patients into diseased and healthy (control) groups (see Supplemental Material, section ‘Chen2015within.R' for preparing this kind of data for analysis). This partitioning is necessary for a statistical correction that also accounts for the agreement between the two tests’ results. While the authors originally used Cohen’s kappa to evaluate agreement, we propose substituting it with Holley & Guilford’s *G* or Gwet’s (AC1).^19^ The present results match those in the original example, showing a null difference of Youden indices of the ELISA and ELISPOT diagnostic methods (Table 3, lower panel) – observe that zero is included in the confidence interval, which corresponds to *p >* 0*.*05.

Another selected example of a within-group design, based on the results of Recker et al. (1998),^26^ aimed to improve PSA accuracy by using the ratio of free to total PSA. The authors applied total PSA (tPSA) tests with the traditional cutoff of 4 ng/mL to 69 patients with cancer and 219 with benign prostate hyperplasia, yielding sensitivity, specificity, and Positive Predictive Value (PPV) of 88 %, 57 %, and 40 %, respectively. They then replaced tPSA with the free/total PSA ratio, reporting changes in sensitivity, specificity, and PPV with thresholds of 0.20 (88 %, 66 %, and 45 %) and 0.15 (70 %, 82 %, and 55 %). Based on these point estimates, the authors claim improvements in one or more of these indices.

Since there is no information on the partition into diseased and control groups required for the agreement correction proposed by Chen et al.,^2^ the function implemented in the eiras2x2 package automatically tries all possible 2 × 2 tables with the available data and compares the most extreme cases. If these extremes reach the same conclusion, the authors can assume that the statistical conclusion applies to the original data, which likely falls between these extremes.

It is shown that, contrary to the authors’ conclusions, there is no statistical difference between the tests, considering a within-group design. In all three versions, these are clearly low-accuracy tests (Table 6).

**Table 6.** Performance difference (within-group design) with data reproduced from Recker et al. (1998) evaluated by the Youden index (*J*) of three Prostate-Specific Antigen (PSA) tests: total PSA (tPSA), free to total PSA with a cut-off point at 0.20 (f/tPSA020), and free to total PSA with a cut- off point at 0.15 (f/tPSA015). 95 % confidence intervals and *p* values were computed using the R function *twosample.Youden* testing $H_{0}:\;J_{2}-J_{1}\;=\;0$. *GD_+_* and *GD_-_* are agreement estimates obtained from Gwet’s AC_1_ (see text for explanation).

			**tPSA**	**f/tPSA0.20**
			*J* = 0.45940	*J* = 0.54616
**f/tPSA0.20**	*J* = 0.54616	min(*GD*_+_)	0.7080	
		min(*GD_-_*)	−0.4440	
		Diff. *J*	0.087	
			[−0.015, 0.188]	
			***p*****=0.0945**	
		min(*GD_+_*)	0.7080	
		max(*GD_-_*)	0.8360	
		Diff. *J*	0.087	
			[−0.010, 0.183]	
			***p*****=0.0775**	
		max(*GD_+_*)	1.0000	
		min(*GD_-_*)	−0.4440	
		Diff. *J*	0.087	
			[−0.013, 0.187]	
			***p*****=0.0890**	
		max(*GD_+_*)	1.0000	
		max(*GD_-_*)	0.8360	
		Diff. *J*	0.087	
			[−0.008, 0.181]	
			***p*****=0.0724**	
**f/tPSA0.15**	*J* = 0.51757	min(*GD_+_*)	0.3710	0.3710
		min(*GD_-_*)	−0.0410	0.1640
		Diff. *J*	0.058	−0.029
			[−0.059, 0.175]	[−0.147, 0.089]
			***p*****=0.3307**	***p*****=0.6346**
		min(*GD_+_*)	0.3710	0.3710
		max(*GD_-_*)	0.5740	0.7410
		Diff. *J*	0.058	−0.029
			[−0.057, 0.173]	[−0.145, 0.087]
			***p*****=0.3221**	***p*****=0.6292**
		max(*GD_+_*)	0.7180	0.7180
		min(*GD_-_*)	−0.0410	0.1640
		Diff. *J*	0.058	−0.029
			[−0.056, 0.172]	[−0.144, 0.086]
			***p*****=0.3186**	***p*****=0.6261**
		max(*GD_+_*)	0.7180	0.7180
		max(*GD_-_*)	0.5740	0.7410
		Diff. *J*	0.058	−0.029
			[−0.054, 0.170]	[−0.142, 0.085]
			***p*****=0.3097**	***p*****=0.6204**

### Returning to the independent samples test of Youden indices across different studies

As we were able to reconstruct the 2 × 2 tables for the two examples above,^25^^,^^26^ and since the outcome under investigation is the same - prostate cancer - the present method allows us to verify whether there is any performance advantage of one diagnostic test over another when tested in pairs. We found no evidence of a performance difference between these methods, despite the 22-year gap between the publications (Table 7).

**Table 7.** Performance difference (between-group design) with data reproduced from Erdogan et al. (2020) and Recker et al. (1998) evaluated by the Youden index (*J*). Erdogan proposed the Prostate Volume (PV) as a predictor of cancer in two groups of patients with two different cut-off points (PSA 2.5‒10 ng/mL with cutoff of PV=43.5 mL; PSA 10.1‒30.0 ng/mL with cutoff of PV=63.5 mL). Recker applied three Prostate-Specific Antigen (PSA) tests: total PSA (tPSA), free to total PSA with a cut-off point at 0.20 (f/tPSA0.20), and free to total PSA with a cut-off point at 0.15 (f/tPSA0.15). 95 % confidence intervals of *J* and *p*-values were computed using the R function *twosample.Youden* testing $H_{0}:\;J_{2}-J_{1}\;=\;0$.

		**PV 43.5 mL**	**PV 61.5 mL**
		*J_1_* = 0.46875	*J_1_* = 0.56379
**tPSA**	*J*_2_ - *J*_1_	−0.00935	−0.10439
*J_2_* = 0.45940		[−0.15514, 0.13644]	[−0.27652, 0.06774]
		***p*****=0.9000**	***p*****=0.2346**
**f/tPSA0.20**	*J*_2_ - *J*_1_	0.07741	−0.01763
*J_2_* = 0.54616		[−0.06951, 0.22433]	[−0.19072, 0.15546]
		***p*****=0.3018**	***p*****=0.8418**
**f/tPSA0.15**	*J*_2_ - *J*_1_	0.04882	−0.04622[−0.22951, 0.13707]
*J_2_* = 0.51757		[−0.10999, 0.20763]	[−0.22951, 0.13707]
		***p*****=0.5468**	***p*****=0.6211**

### Assessing other biomarkers

To compare PSA with novel or emerging biomarkers using the same statistical rigor and to highlight incremental benefits or shortcomings, we searched for published studies that provided sufficient information to extract data and generate 2 × 2 contingency tables. This is not intended to offer a definitive answer to the complex clinical challenges related to prostate cancer, but rather to illustrate how the Youden index can be applied to assess whether proposed advances (using prostate cancer as an example) are statistically sound for both current and future diagnostic tests.

Among emerging biomarkers, urinary PCA3 was evaluated by Deras et al. (2008) in a multicenter study with 570 men undergoing initial or repeat prostate biopsy, showing consistent performance across PSA subgroups.^27^ In a smaller study in Chile, Ramos et al. (2013) also reported its superiority over traditional PSA.^28^ Multiparametric MRI was assessed by Thompson et al. (2014) in 223 biopsy-naïve men and found to outperform standard methods in detecting clinically significant prostate cancer.^29^ Al Saidi et al. (2017) compared PHI and %p2PSA in 136 men, reporting better accuracy for PHI.^30^ SelectMDx, a risk model based on combinations of urinary biomarkers designed to detect high-grade prostate cancer, was evaluated by Van Neste et al. (2016) in a 386-man cohort and in a 14 study meta-analysis by Wu et al. (2024) with a total of 2579 patients, concluding that this test has moderate to good diagnostic accuracy in distinguishing clinically significant prostate cancer among high- risk patients, reducing unnecessary biopsies.^31^^,^^32^ Parekh et al. (2015) assessed the 4Kscore in 1012 men from 26 centers, also concluding that it could reduce unnecessary biopsies while preserving detection of aggressive disease.^33^ Finally, Derderian et al. (2022) proposed a liquid biopsy approach using a 14-gene expression panel from blood RNA; while promising, their study was preliminary and based on a small sample.^34^

Table 8 compares the proposed biomarkers with total PSA (tPSA), using Recker et al. (1998)^26^ as a reference. Statistical significance in this table is shown in two columns of p-values. The first refers to the 95 % Confidence Interval of the test itself, indicating that some do not even qualify as diagnostic tests, thus, assessing whether they represent an improvement over tPSA is meaningless. Interestingly, these failed exams include tPSA in samples from Ramos et al. (2013)^28^ and Saidi et al. (2017),^30^ as well as applications of PCA3 by Ramos et al.(2013) when there is either no prior biopsy or a prior negative biopsy, and the biomarkers TDRD1 and DLX1 proposed by Van Neste et al. (2016).^31^ For the remaining biomarkers, the second *p*-value column refers to the 95 % Confidence Interval of the difference from the reference Youden index (*Diff. J*). When a significant difference is observed, one may consider it progress if the difference is positive, which occurred only with PHI evaluated by Al Saidi et al. (2017) and the Liquid Biopsy tested by Derderian et al. (2022).^34^ In the other cases, the difference was either non-significant or negative, indicating worse performance than the conventional PSA test.

**Table 8.** Comparison of Recker et al.’s total PSA (reference) with proposed biomarkers. Statistical significance is highlighted with *p* values in **boldface**. When a proposed biomarker qualifies as a diagnostic test (95 % confidence interval for *J* is entirely above zero), advance is considered if the difference from the reference (*Diff. J*) is also significantly greater than zero.

**a**	**b**	**c**	**d**	***J***		***Diff. J* = *J***_*ref*_***− J***	
						
**Recker et al., 1998**				**(*****J**_**ref**_***)**			
tPSA: 61	93	8	126	0.4594 [0.3987, 0.5201]	***p*****=7.87 ⋅ 10***^**–**^*^**36**^	r**eference**	
**Deras et al., 2008**							
PCA3 overall: 112	92	96	262	0.2786 [0.2317, 0.3254]	***p*****=7.14 ⋅ 10***^**–**^*^**23**^	−0.1808 [−0.2722, −0.0894]	***p*****=1.06⋅ 10***^**–**^*^**04**^
PCA3 (PSA *<* 4): 17	22	17	75	0.2732 [0.1587, 0.3877]	***p*****=4.36 ⋅ 10***^**–**^*^**05**^	−0.1862 [−0.3407, −0.0317]	***p*****=0.0181**
PCA3 (4 *<* PSA *<* 10): 69	62	62	153	0.2383 [0.1807, 0.2960]	***p*****=5.22 · 10***^**–**^*^**12**^	−0.2211 [−0.3208, −0.1213]	***p*****=1.41⋅ 10***^**–**^*^**05**^
PCA3 (PSA *>* 10): 25	9	16	36	0.4098 [0.2930, 0.5265]	***p*****=3.87 ⋅ 10***^**–**^*^**09**^	−0.0496 [−0.2064, 0.1072]	*p* = 0.5349
**Ramos et al. 2013**							
tPSA: 19	23	4	6	0.0330 [−0.0727, 0.1387]	*p* = 0.3039	−0.4264 [−0.5717, −0.2812]	Meaningless
PCA3: 12	4	11	25	0.3838 [0.2262, 0.5414]	***p*****=3.09 ⋅ 10***^**–**^*^**05**^	−0.0756 [−0.2768, 0.1256]	*p* = 0.4616
	tPSA with previous negative biopsy:						
8	5	0	2	0.2857 [0.0049, 0.5666]	***p*****=0.0471**	0.1737 [−0.5161, 0.1687	*p* = 0.3201
	PCA3 with previous negative biopsy:						
3	1	5	6	0.2321 [−0.0226, 0.4869]	*p* = 0.0669	−0.2273 [−0.5393, 0.0848]	Meaningless
	tPSA without previous biopsy:						
19	18	5	6	0.0417 [−0.0784, 0.1617]	*p* = 0*.*2840	−0.4177 [−0.5780, −0.2574]	Meaningless
	PCA3 without previous biopsy:						
14	3	10	21	0.4583 [0.2997, 0.6170]	***p*****=1*****.*****01 ⋅ 10***^**–**^*^**06**^	−0.0011 [−0.2035, 0.2014]	*p* = 0.9918
**Thompson et al., 2014**							
	Scenario 1: (more strict grade only, Gleason score ≥ 4 + 3):						
70	37	5	38	0.4400 [0.3482, 0.5318]	***p*****=1.61 ⋅ 10***^**–**^*^**15**^	0.0194 [−0.1506, 0.1118]	*p* = 0*.*7719
	Scenario 2: (less strict grade only, Gleason score ≥ 3 + 4):						
72	40	3	35	0.4267 [0.3341, 0.5193]	***p*****=1.75 ⋅ 10**^**–14**^	−0.0327 [−0.1647, 0.0992]	*p* = 0.6269
	Scenario 3: (more strict grade + volume, Gleason score ≥ 4 + 3 or *>* 50 % core involvement):						
70	35	4	40	0.4793 [0.3867, 0.5718]	***p*****=8.12 ⋅ 10***^**–**^*^**18**^	0.0199 [−0.1120, 0.1518]	*p* = 0.7677
	Scenario 4: (less strict grade + volume, Gleason score ≥ 3 + 4 or *>* 33 % core involvement):						
72	37	3	38	0.4667 [0.3736, 0.5598]	***p*****=8.20 ⋅ 10**^**–17**^	0.0073 [−0.1252, 0.1397]	*p* = 0.9144
**Saidi et al. 2017**							
tPSA: 22	80	6	28	0.0450 [−0.0188, 0.1087]	*p* = 0.1229	−0.4144 [−0.5193, −0.3095]	Meaningless
PHI: 23	21	5	87	0.6270 [0.5181, 0.7358]	***p*****=1.32 ⋅ 10**^**–21**^	0.1676 [0.0191, 0.3161]	***p*****=0.0270**
%p2PSA: 18	19	10	89	0.4669 [0.3382, 0.5956]	***p*****=1.21 ⋅ 10**^**–09**^	0.0075 [−0.1621, 0.1771]	*p* = 0.9306
**Van Neste et al., 2016**							
PCA3: 448	309	44	77	0.1101 [0.0793, 0.1408]	***p*****=1.98 ⋅ 10***^**–**^*^**09**^	−0.3493 [−0.4305, −0.2682]	***p* ≪ 0.0001**
TDRD1: 443	343	49	43	0.0118 [−0.0120, 0.0357]	*p* = 0.2077	−0.4476 [−0.5253, −0.3698]	Meaningless
DLX1: 408	324	84	62	−0.0101 [−0.0360, 0.0158]	*p* = 0.2603	−0.4695 [−0.5482, −0.3908]	Meaningless
HOXC4: 448	301	44	85	0.1308 [0.0988, 0.1627]	***p*****=8.12 ⋅ 10***^**–**^*^**12**^	−0.3286 [−0.4104, −0.2469]	***p*****=3.33 ⋅ 10***^**–**^*^**15**^
HOXC6: 448	259	44	127	0.2396 [0.2031, 0.2761]	***p*****=1.72 ⋅ 10***^**–**^*^**27**^	−0.2198 [−0.3042, −0.1354]	***p*****=3.35 ⋅ 10***^**–**^*^**07**^
	HOXC4 and DLX1:						
448	267	44	119	0.2189 [0.1831, 0.2547]	***p*****=4.38 ⋅ 10***^**–**^*^**24**^	−0.2405 [−0.3246, −0.1565]	***p*****=2.01 ⋅ 10***^**–**^*^**08**^
	HOXC4 and TDRD1:						
448	270	44	116	0.2111 [0.1756, 0.2466]	***p*****=7.32 ⋅ 10***^**–**^*^**23**^	−0.2483 [−0.3322,−0.1645]	***p*****=6.46 ⋅ 10***^**–**^*^**09**^
	HOXC4, DLX1, and TDRD1:						
448	267	44	119	0.2189 [0.1831, 0.2547]	***p*****=4.38 ⋅ 10***^**–**^*^**24**^	−0.2405 [−0.3246, −0.1565]	***p*****=2.01 ⋅ 10***^**–**^*^**08**^
	HOXC6 and DLX1:						
448	247	44	139	0.2707 [0.2333, 0.3081]	***p*****=5.20 ⋅ 10***^**–**^*^**33**^	−0.1887 [−0.2737, −0.1037]	***p*****=1.35 ⋅ 10***^**–**^*^**05**^
	HOXC6 and TDRD1:						
448	251	44	135	0.2603 [0.2232, 0.2974]	***p*****=4.11 ⋅ 10***^**–**^*^**31**^	−0.1991 [−0.2839, −0.1143]	***p*****=4.21 ⋅ 10***^**–**^*^**06**^
	HOXC6, DLX1, and TDRD1:						
448	254	44	132	0.2525 [0.2157, 0.2894]	***p*****=9.96 ⋅ 10***^**–**^*^**30**^	−0.2069 [−0.2915, −0.1222]	***p*****=1.68 ⋅ 10***^**–**^*^**06**^
	HOXC6, HOXC4, DLX1, and TDRD1:						
448	259	44	127	0.2396 [0.2031, 0.2761]	***p*****=1.72 ⋅ 10***^**–**^*^**27**^	−0.2198 [−0.3042, −0.1354]	***p*****=3.35 ⋅ 10***^**–**^*^**07**^
**Wu et al., 2023**							
SelectMDx: 941	681	220	737	0.3303 [0.3100, 0.3505]	***p*****=5.15 ⋅ 10***^**–**^*^**159**^	−0.1291 [−0.2054, −0.0529]	***p*****=0.0009**
**Parekh et al., 2021**							
4KScore: 207	371	24	410	0.4211 [0.3895, 0.4526]	***p*****=3.08 ⋅ 10***^**–**^*^**107**^	−0.0383 [−0.1199, 0.0432]	*p* = 0.3569
**Derderian et al., 2022**							
	Liquid Biopsy, risk classification from 14-gene panel:						
17	0	3	48	0.8500 [0.7187, 0.9813]	***p*****=9.12 ⋅ 10***^**–**^*^**27**^	0.3906 [0.21820, 0.5630]	***p*****=8.99 ⋅ 10***^**–**^*^**6**^

## Discussion

This work is based on the Youden index, which has many alternative formulas,^35^^,^^36^ but it is easy to remember J=se+sp−1. This leads to a rule of thumb: se+sp>1, allowing physicians to sum sensitivity and specificity. If this sum exceeds 1, the closer it is to 2, the better the test quality. Since this heuristic is not infallible, it is recommended to complement it with statistical tests.

The first test uses the Youden index to determine whether an examination qualifies as a diagnostic test (*J >* 0). The second compares two tests to assess performance differences, with the aim of improving or replacing them, considering both within-group (the same patients) and between-group (different patients) evaluations. These focus on test quality.

Diagnosis quality, however, depends on disease prevalence or the physician’s estimate of pre-test probability. Examples show how diagnostic tests function in both nomothetic (epidemiological) and idiographic (clinical) contexts.

**Fig. 3:**
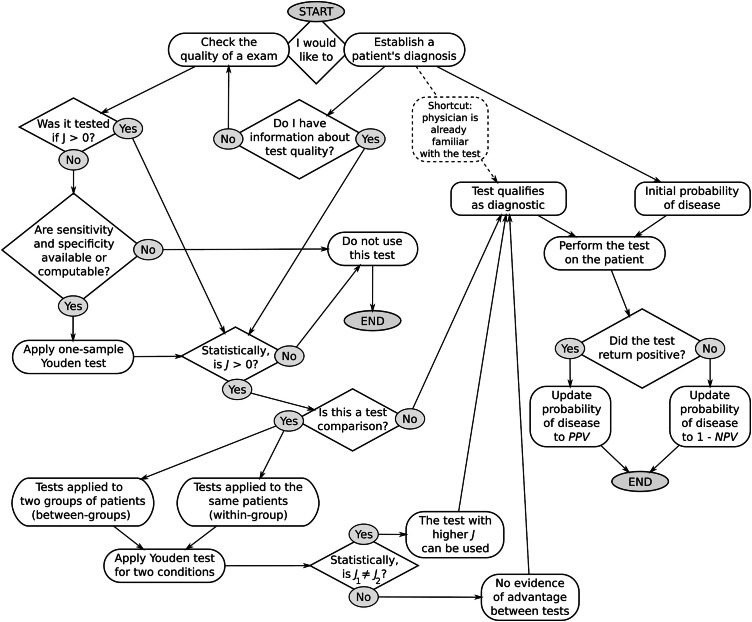
Decision-making algorithm integrating Youden index (*J*) with prevalence-adjusted positive (*PPV*) and negative (*NPV*) predictive values.

There are pitfalls in assuming that diagnostic tests are interpretable without understanding the interaction of sensitivity, specificity, and disease probabilities. Here, we demonstrate that (1) The Youden index is useful to assess test quality; (2) Diagnosis exclusion relies more on sensitivity, while confirmation depends on specificity (Fig. 2A and B); (3) Tests with high sensitivity and specificity can still result in a low probability of disease despite positive results, as shown in Fig. 2C; and (4) Attempts to improve PSA and its variants for detecting prostate cancer are statistically equivalent, with performance remaining mediocre (Fig. 2D, E, and F).

To support practical use of this method, a decision-making flowchart was included, integrating the Youden index with predictive values (Fig. 3). Though initially complex, it summarizes the manuscript’s logic, covering test evaluation and individual diagnosis. It highlights two perspectives: researchers verifying test improvements and clinicians applying the Youden index with prevalence-adjusted PPV/NPV for patient diagnosis. The figure shows two complementary paths: the left branch guides evaluating test quality by verifying *J >* 0 with one-sample tests or comparing *J*_1_ ≠ *J*_2_ using two-condition Youden tests (within- or between-group designs). The right branch focuses on applying a validated test to update disease probability in individual patients using *PPV* or 1–*NPV*.

Many studies claiming improvements omit raw data, making it hard to reconstruct 2 × 2 tables for verification. ROC curve analyses comparing AUCs also suffer from limited raw data access, hindering independent checks. In contrast, the Youden index requires only the contingency table, which is more often available in published reports.

As seen in Siegel et al. (2010, Fig. 3, page 16),^3^ cancer incidence rates change slowly over time, except for prostate cancer. A notable peak in prostate cancer cases was observed between 1990 and 2013, coinciding with the widespread adoption of PSA testing and improved diagnostic techniques.^37^ PSA testing, introduced in the late 1980s and expanded in the early 1990s, likely identified many indolent cases, contributing to potential overdiagnosis.^38^^,^^39^ Advances such as transrectal ultrasound and needle biopsies also increased detection during this period.^40^ Following the peak, incidence rates declined and stabilized by 2013 as screening became more conservative.^3^^,^^37^^,^^41^ In Brazil, a steady decline in PSA screening has been noted,^42^ likely due to updated guidelines discouraging its use.^43^ Despite this, prostate cancer-specific mortality rates have plateaued. Limited data collection in less developed regions, including Brazil and Latin America, along with varying screening recommendations, may prompt critical discussions.^44^

Contrary to this trend, a recent Brazilian Ministry of Health (BMH) guideline advises that “men over 45 with risk factors or over 50 without should consult a urologist to discuss digital rectal exams and PSA tests”.^10^ The Federal Unified Health System (SUS) provides free access to these tests for the population,^11^ while a public booklet notes that “some specialists oppose and others support routine exams for asymptomatic men due to potential benefits and risks”.^12^ Among them, the National Institute of Cancer (INCA) issued a technical note advising against population-wide prostate cancer screening.^13^ Despite INCA’s stance, no clear decision has been made about suspending the up to 2024 campaign, leaving uncertainty as the BMH remains non-committal.

Even as early detection using PSA combined with risk calculators and MRI may improve follow-up,^45^ statistics indicate a plateau in mortality rates, seemingly following reduced PSA screening,^46^ as recommended by the US Preventive Services Task Force (USPSTF) in 2012.^47^ However, the American Urological Association (AUA) and Society of Urologic Oncology (SUO) still support PSA screening with shared decision-making.^48^ Two major studies assessed screening’s impact: the North American study showed no overall survival benefit, while the European study found a 35 % reduction in deaths. The discrepancy is linked to 50 % of the American control group receiving routine PSA tests, a methodological issue.^49^

*J*-based evaluation offers a robust, prevalence-independent measure for test selection and cutoff optimization, while prevalence-adjusted PPV/NPV contextualize performance for specific populations. Together, they guide evidence-based, regularly updated protocols for urology societies (AUA/EAU), ensuring clinical relevance and efficient resource use, especially in low-prevalence or resource-limited settings.

Overdiagnosis and underdiagnosis pose ethical challenges, causing patient harm (unnecessary procedures, distress, financial burden) and societal issues (resource misallocation, inequity). Mitigation strategies include evidence-based practices, policy reforms, and ethical frameworks like shared decision-making, reducing low-value tests via financial disincentives, and tightening diagnostic criteria. One study estimates a lifetime false-positive risk of up to 85.5 % among baseline women and 38.9 % among baseline men across multiple screening programs, higher in frequently screened groups.^50^ The validation approach here aligns with predictive models and biomarker panels.^51^, ^52^, ^53^

PSA alone is insufficient for prostate cancer screening, highlighting the need for better diagnostic tests. Many biomarker studies suffer from poor biostatistics and methodological flaws, undermining reliability and reproducibility.^54^ Despite a meta-analysis (*n* = 12,781), strong evidence for decision aids in screening remains lacking.^55^ The WHO’s endorsement of PSA testing as a recommendation “grounded in substantial evidence but recognizing its limitations” exemplifies this dilemma. Policymakers and scientists advocate targeted, individualized PSA use combined with shared decision-making to maximize benefits and minimize harms.^56^

This work’s core message is that, beyond showing PSA as a weak diagnostic tool, the proposed statistical method effectively measures how much better new or improved tests are compared to existing ones. This evaluation strengthens clinical decisions and improves patient care. Using PSA as an example, the method can be applied to assess any new diagnostic improvements.

## Data availability

Data and R scripts to replicate statistical tests, figures, and tables are available in Harvard Dataverse at https://doi.org/10.7910/DVN/5QTMBW.

## Ethics

In this study, the use of secondary data sources exempts the research from requiring ethical approval by a review board. The data used were previously collected and are publicly available, ensuring that no new data collection or interaction with the participants occurred and no identifiable information about the subjects was used in the current analysis.

## Declaration of generative AI and AI-assisted technologies in the writing process

Overleaf was used to automate bibliographic formatting (with references curated via Mendeley), as well as the numbering of figures, tables, and citations, in order to avoid the classic pitfalls of manual editing ‒ before the content was exported to Word. Microsoft Word and Excel, as well as the R and JavaScript languages, were employed for writing, data handling, and programming. All content is original, fully authored, reviewed, and edited by the authors, who take full intellectual responsibility for the content of the publication.

## Authors’ contributions

Conceptualization: PSPS, JOS. Data curation: not applicable, secondary data. Formal analysis: PSPS, JOS. Investigation: PSPS, FTR, JEV, JOS. Methodology: PSPS, JOS. Software: JOS, PSPS. Validation: PSPS, FTR, JEV, JOS. Visualization: JOS, PSPS. Writing-original draft: PSPS, FTR, JEV, JOS. Writing-review and editing: PSPS, FTR, JEV, JOS. FTR and JEV are responsible for the contextualization of this research. PSPS developed and prepared the R scripts. JOS developed and implemented the statistical parameterization with the help of PSPS. All authors collaborated in proposing the basic issue and reviewed the results to reach a consensus.

## Funding

This research received no specific grant from funding agencies in the public, commercial, or not-for-profit sectors.

## Declaration of competing interest

The authors declare that there is no conflict of interest with respect to the publication of this manuscript. All authors have approved the final version of the manuscript and agree with its submission. The authors have no affiliations with or involvement in any organization or entity with any financial or nonfinancial interest in the subject matter or materials discussed in this manuscript.
